# VSA-3000: A Quantitative Vibration Sensation Testing Device for Patients With Central Nervous System Injury

**DOI:** 10.3389/fneur.2020.00936

**Published:** 2020-09-08

**Authors:** Mingming Gao, Xiaoping Yun, Tong Zhang

**Affiliations:** ^1^School of Rehabilitation Medicine, Capital Medical University, Beijing, China; ^2^Department of Rehabilitation Evaluation, China Rehabilitation Research Center, Beijing Bo'ai Hospital, Beijing, China; ^3^Neurorehabilitation Center, China Rehabilitation Research Center, Beijing Bo'ai Hospital, Beijing, China

**Keywords:** VSA-3000, vibration perception thresholds, quantitative sensory testing, central nervous system, stroke, spinal cord injury

## Abstract

**Objective:** To investigate the effect of using Vibration Sensory Analyzer-3000 (VSA-3000) in patients with impaired vibration sensation caused by central nervous system injury.

**Design:** Prospective observational study.

**Setting:** A university hospital for the research and clinical practice of rehabilitation.

**Subjects:** Sixty patients (30 stroke and 30 spinal cord injury) were recruited, aged between 20 and 71 years old, under stable medication.

**Interventions:** Not applicable.

**Main Measure:** VSA-3000 threshold test, tuning fork test and somatosensory evoked potential (SSEP) measurement.

**Results:** Test-retest reliability was determined based on data collected from 60 subjects, and the intraclass correlation coefficient (ICC) for vibration perception thresholds (VPTs) was in the “substantial” range. The kappa value between VSA-3000 and SSEP was 0.877, which was higher than that of tuning fork (κ = 0.732). VSA-3000 had good diagnostic accuracy with a sensitivity of 94.8%, specificity of 92.9%, and positive-predictive value of 93.8% and negative-predictive value of 94.0%, each value was higher than that of tuning fork. The area under the receiver operating characteristic curve (AUC) of VSA-3000 was 0.95 (95% CI: 0.91 to 0.98) and that of tuning fork was 0.89 (95% CI: 0.85 to 0.95), and there was a significant difference between the two values (*P* = 0.0216). The types of injury and age were the independent correlates of the VPTs.

**Conclusion:** The present study provides preliminary evidence that VSA-3000 is a non-invasive and convenient quantitative testing instrument with good diagnostic accuracy, and it may be useful as a screening tool for assessing impaired vibration sensation caused by central nerve injury.

## Introduction

Symptoms of proprioceptive disorder are common in diseases of the central nervous system (CNS). Previous studies indicated that in 70 first stroke patients, 34–64% had impaired proprioception (1). About 50–80% of patients with spinal cord injury (SCI) have pressure ulcers caused by sensory (including proprioception) loss (2). In particular, diminished vibration sensation is an important finding in the diagnosis of disorders affecting the dorsal column-medial lemniscus pathways in the CNS and may also be an early sign of CNS diseases (3).

Traditionally, a tuning fork is used for the evaluation of the vibration sense in patients with CNS diseases. This simple instrument has the advantage of being economical, portable, and quick for gross assessment of the sensory system (4), but unfortunately does not quantitatively provide the degree of dysfunction of vibration sense. It is of clinical importance that the vibration sense should be measured quantitatively and consistently. For this purpose, electrophysiology tests have been developed (5, 6), but they are invasive, time consuming, expensive, non-portable and requires a high standard of training to perform (4).

Recently vibration perception threshold (VPT) by quantitative sensory testing (QST) has been proposed as a method to assess the somatosensory pathways in clinical trials (7, 8). Multiple studies showed that VPT was a sensitive measure of peripheral neuropathy (9–17). The QST method for measuring VPTs has shown higher reliability than the tuning fork testing (7). Meanwhile it is painless and only requires brief training in comparison with electrophysiological testing (7, 18).

As one of QST computerized devices, the Vibration Sensory Analyzer VSA-3000 (Medoc) was designed to assess vibration. VPT assessed by VSA-3000 has been most commonly used in detecting peripheral neuropathy (9, 12–14, 19–23). Recent studies showed that QST using VSA-3000 (or other devices) was also a useful adjunct measurement with good reliability of detection thresholds in central nervous system diseases (6, 24–28). However a specific analysis of its diagnostic accuracy with VSA-3000, especially as diagnostic outcome measures in patients with stroke and SCI, has not been fully established.

Therefore, this study has two aims: (1) to estimate the diagnostic accuracy of the QST using VSA-3000 in evaluating VPT, in patients with CNS injury, against the reference standard of somatosensory evoked potential (SSEP) measurements, and (2) to assess whether the VSA-3000 device offers superior accuracy compared with other routine test (e.g., the tuning fork) for impaired vibration sensation caused by CNS diseases.

## Methods

### Subjects

Individuals with stroke and SCI were recruited through advertisements posted at China Rehabilitation Research Center (CRRC) and Capital Medical University School of Rehabilitation Medicine, and by word of mouth (from May 2015 to March 2018). The study was approved by the Ethical Committee of CRRC.

Participants had to be: (a) age 18 years or older; (b) first-ever stroke (29) patients with unilateral sensory disturbance and with lesions in basal ganglia detected on radiological means, or patients with a thoracic or lumbar SCI; (c) medically stable conditions (patients' disease has not progressed within 1 week), ability to give informed consent and understand and cooperate with the testing. The exclusion criteria were presence of diabetes or other diseases involving neurologic impairments.

### General Protocol

Subjects with stroke and SCI who met the inclusion criteria were scheduled for their first study visit. After informed consent was obtained, a neurological examination was conducted and a second visit was scheduled. During the second visit, three types of measurements (VSA-3000, tuning fork and SSEP) were conducted. Sixty participants (30 stroke and 30 SCI) in all completed an identical VSA-3000 test session ~1 to 4 weeks later to provide data for the test-retest analysis portion of the present study.

### Clinical Characteristics

Each participant's age, height, course of disease, and sex were recorded in the interview. For SCI patients, additional questions regarding the cause of injury were included [falls 14 (46.7%), violence 7 (23.3%), vehicle crashes 5 (16.7%), and others 4 (13.3%)]. For each participant with stroke, an experienced physician conducted a physical examination, to assess neurological status and to diagnose the type of the stroke according to the classification of cerebrovascular disorders of World Health Organization (29). For each participant with SCI, a physician with extensive SCI experience conducted a physical examination, including the American Spinal Injury Association (ASIA) standard examination (30), to assess neurological status and determine the severity (complete or incomplete) of injury.

**Figure 1:**
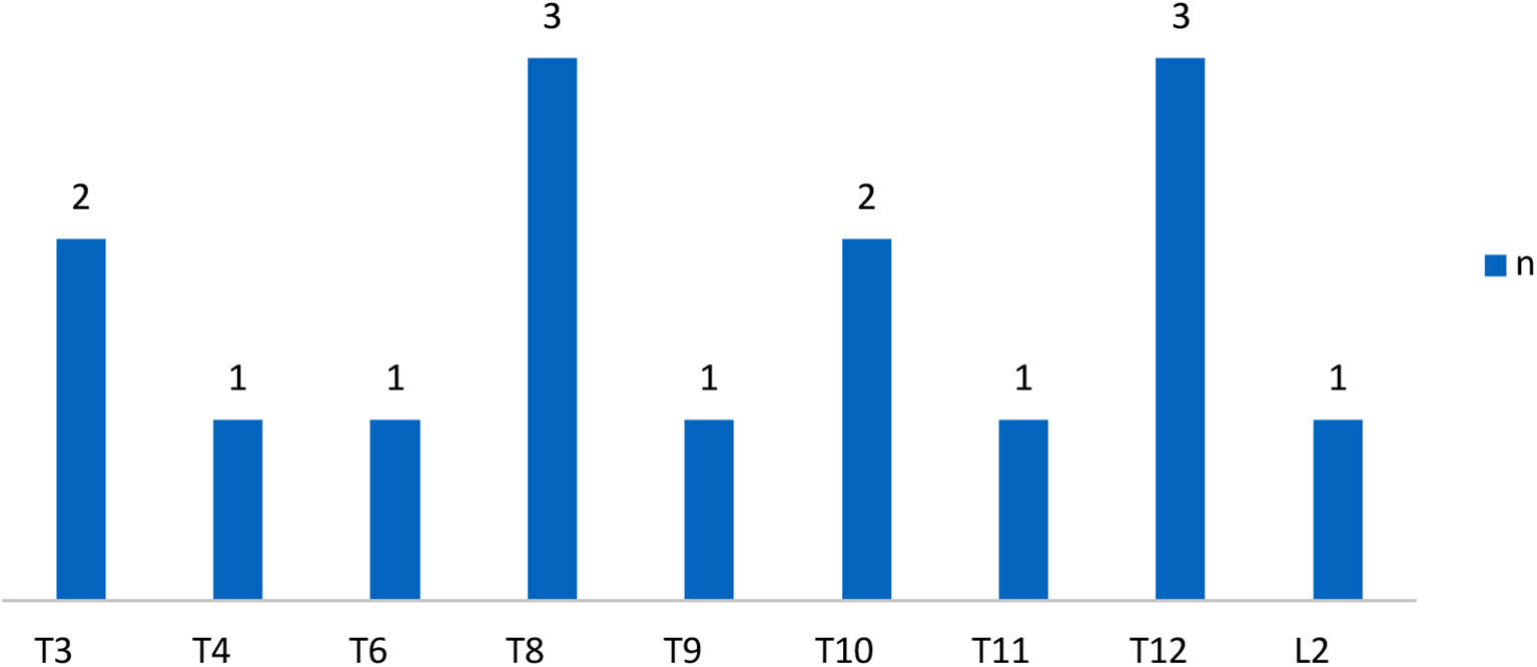
Distribution of neurologic levels of injury in complete SCI patients. T, thoracic levels; L, lumbar levels; (30) n, numbers.

**Figure 2:**
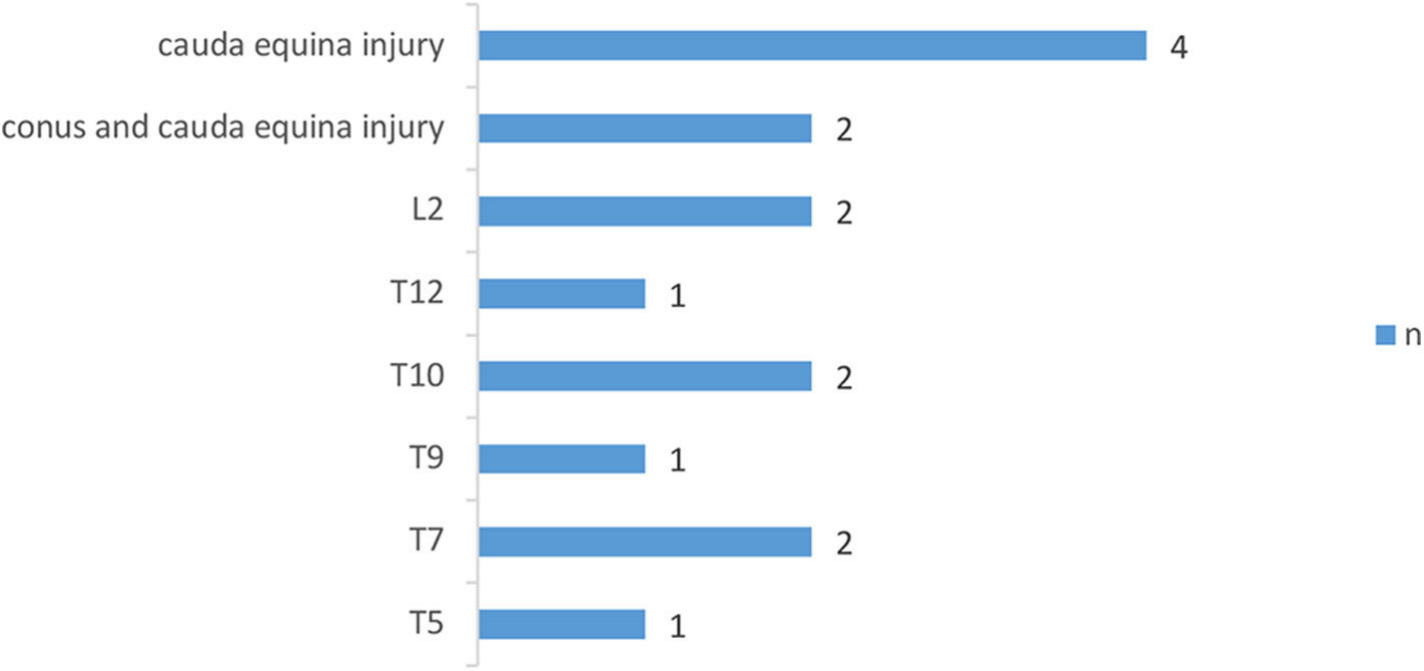
Distribution of neurologic levels of injury in incomplete SCI patients. T, thoracic levels; L, lumbar levels; (30) n, numbers.

The demographic characteristics of subjects were shown in Table 1 and the distribution of neurological level of SCI participants was shown in Figures 1, 2.

**Table 1:** Demographic characteristics of subjects (*n* = 60).

Age (yrs)	43.38 ± 12.98
Height (cm)	169.97 ± 6.49
Course of disease (d)	58.00 (33.25,96.50)
Sex	
Male	50 (83.3)
Female	10 (16.7)
Type	
SCI	
Incomplete	15 (25.0)
Complete	15 (25.0)
Stroke	
Hemorrhage	28 (46.7)
Infarction	2 (3.3)

*Values are mean ± standard deviation, median (P25, P75) or n (%)*.

### Tests

All the tests were performed by experienced physicians in a quiet room with an approximate temperature between 22 and 24°C. Subjects were tested in their own wheelchair to complete the tests of VSA-3000 and tuning fork, and lying prone relaxed for SSEP tests. Before testing, the examiner explained the procedures and several pilots were performed so that subjects could be familiar with the tests.

**Figure 3:**
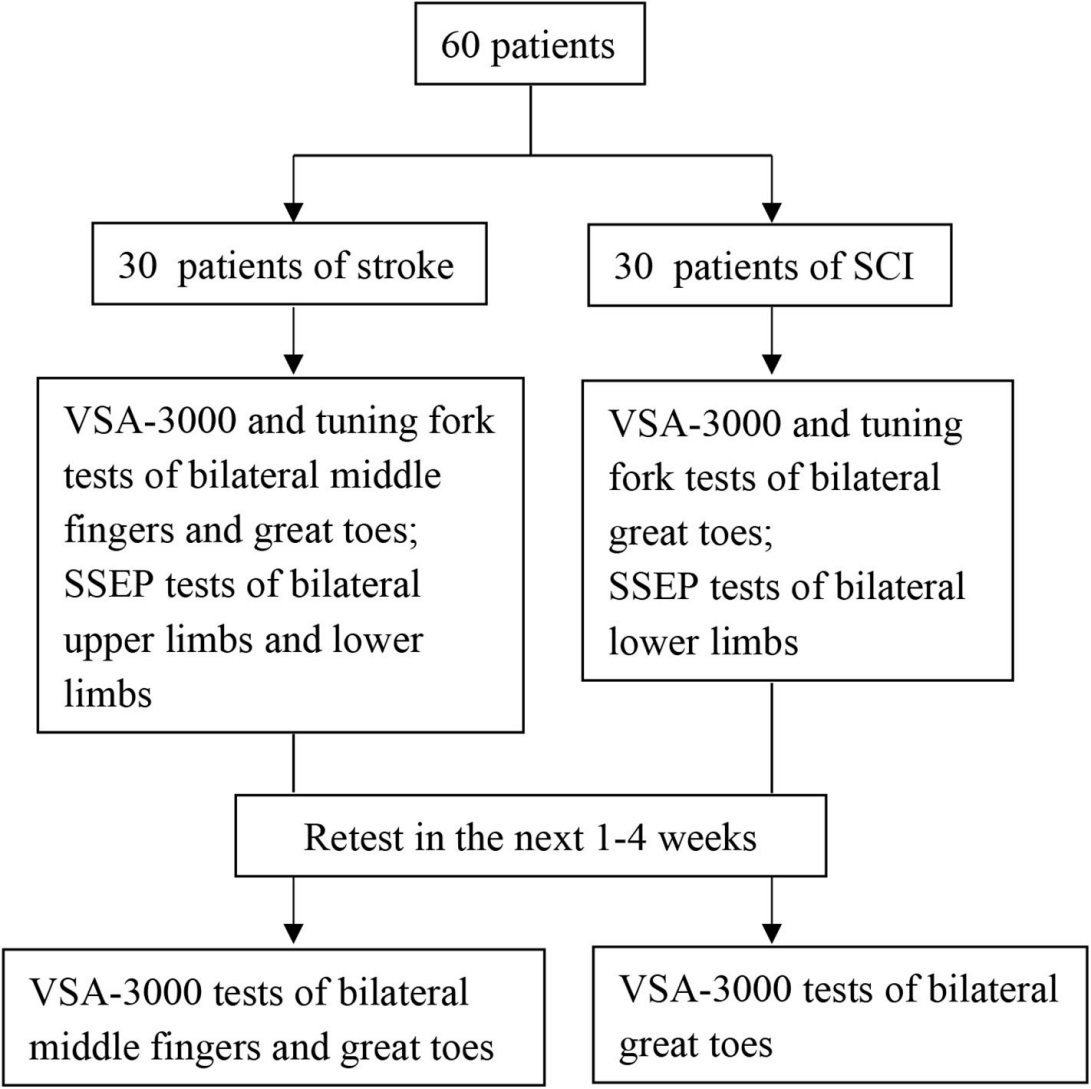
Flow chart of the sequential tests.

Figure 3 outlines the sequential tests.

#### Quantitative Sensory Testing Using VSA-3000

**Figure 4:**
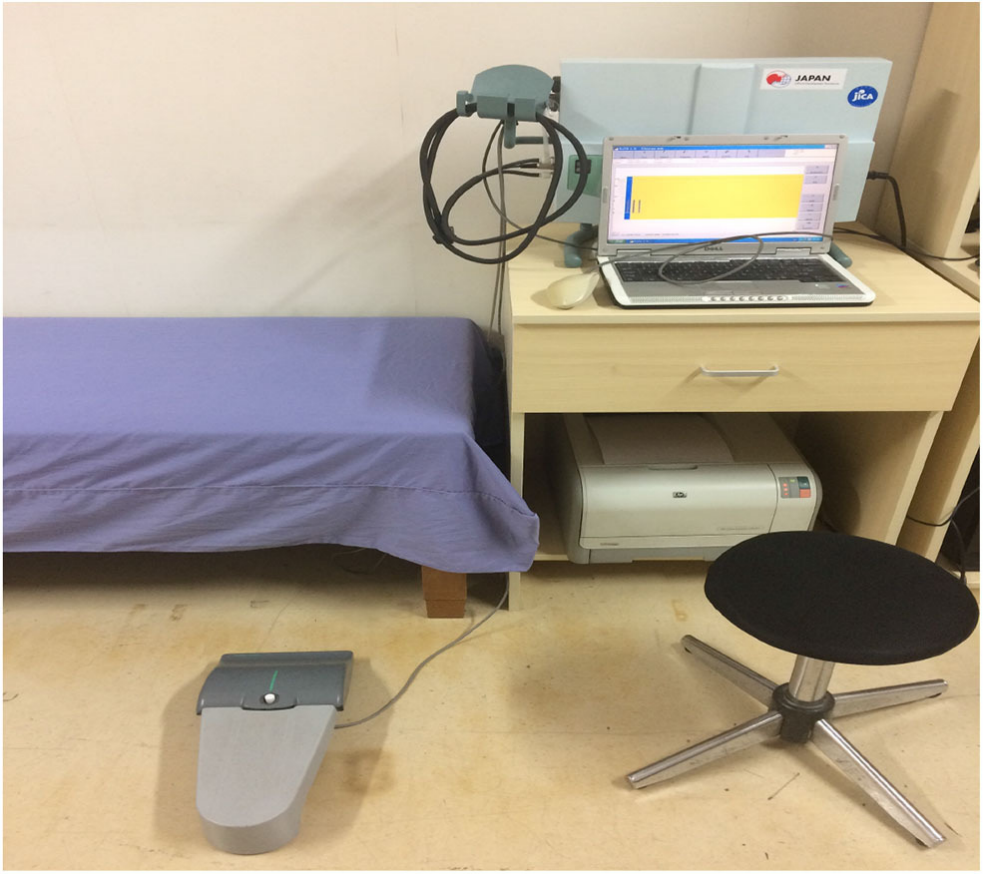
VSA-3000 (Medoc, Israel).

Quantitative VPT was measured using the VSA-3000 vibratory sensory analyzer (Medoc Ltd., Israel) (Figure 4) following published protocols (31). The diameter of the stimulating probe was 1.2 cm and the vibratory stimulus was delivered at 100 Hz. The stimulating surface of the vibratory probe was placed on the hand (the palm side of the middle finger) and the foot (the plantar side of the great toe) (32).

The vibratory thresholds were measured by the method of limits (33). The device delivered the stimulus with increasing intensity starting from the baseline (0 μm) at a rate of 0.8 μm/s (lower limb) or a rate of 0.4 μm/s (upper limb) until the subject indicated that the stimulus was felt or until the maximum amplitude of 130 μm was reached. Subjects were asked to indicate by clicking the mouse as soon as they felt the vibratory sensation. The next trial started again from the baseline value, with the average of three successive trials (separated by 10 s each) (25) taken as the vibration perception threshold (VPT) for each site. To include data for analyses at sites where no sensation was evoked during testing, we recorded the maximum amplitude of the vibratory stimulus (cutoff value) (VPT=130 μm) (25). According to the standard of normal values specified by the VSA-3000 manufacturer, VPTs were divided into three groups (normal, decreased, and undetected) (Table 2).

**Table 2:** Vibration perception thresholds (VPTs) of VSA-3000.

**Age (yrs)**		**20–29**	**30–39**	**40–49**	**50–59**	**60–69**	**70–79**
VPTs of	Normal	0–1.7	0–2	0–2.4	0–3	0–4	0–5.6
middle finger (μm)	Decreased	130≥VPT>1.7	130≥VPT>2	130≥VPT>2.4	130≥VPT>3	130≥VPT>4	130≥VPT>5.6
	Undetected	>130					
VPTs of great toe (μm)	Normal	0-8.2	0-10	0-14	0-22.8	0-43	0-90
	Decreased	130≥VPT>8.2	130≥VPT>10	130≥VPT>14	130≥VPT>22.8	130≥VPT>43	130≥VPT>90
	Undetected	>130					

#### Physical Examination Testing Using 128 Hz Tuning Fork

The vibratory sensation was tested with a 128-Hz tuning fork at the same sites as those in the VSA-3000 test. The examiner energized the tuning fork by fully opposing the two blades together where blades touched each other, rapidly released by slipping the fingers off the blade ends (34), and then immediately placed the base of the tuning fork on the test sites. The first measurements were taken at the palm side of the right middle finger of SCI subjects or at the palm side of the unaffected side middle finger of stroke subjects as a reference vibratory sensation (regarded as normal), then testing progressed to other sites, including the plantar side of bilateral great toes of all subjects and the palm side of the affected side middle fingers of stroke subjects. In this manner, we could ask subjects to compare the quality of the sensation to the quality evoked at the reference hand. Appreciation of vibratory sensation at each site was separately scored on a 0-10 numerical rating scale scale (25, 35), with 0 = “undetected,” 1–9 = “decreased,” 10 = “normal.”

#### Electrophysiology Testing Using Evoked Potential Instrument

Somatosensory evoked potential (SSEP) measurements were performed by a conventional EMG machine (Dantec Keypoint, Denmark). The tibial and median SSEP were elicited by electrical stimulation (square-wave stimulation of 0.2 ms at a frequency of 3 Hz) at the ankle or wrist with the cathodes placed 2 to 3 cm proximal to the anode (36). Stimulus intensity was adjusted to produce a clear muscular response (max 30 mA) in order to assess all sensory fibers (37).

According to the international nomenclature, in the waveforms of SSEP, positive peaks are represented by downward deflections and labeled P and negative peaks are represented by upward deflections and labeled N (38). The lower limb response elicited by electrical stimulation of the tibial nerve has a main positive peak with a latency of ~40 ms labeled as P40, and the upper limb response elicited by electrical stimulation of the median nerve has a main negative peak with a latency of ~20 ms labeled as N20.

For recording, scalp electrodes (0.5 cm silver plate electrodes) were applied at Cz′/Fz and C3′/C4′/Fz using the International 10/20 electrode system (39). The electrode impedance was maintained below 5 kΩ. The amplifier was set at 5 μv/division, frequency bandpass was set at 30–3,000 Hz. Three sets of 200 responses were averaged and superimposed to ensure consistency. The P40 and N20 latencies were recorded and used for statistical analysis.

## Statistical Analysis

Test-retest reliability, a measure of the stability of a test when it is administered across time without changes in other variables, was evaluated separately for SCI and stroke subjects for VSA-3000 test by using intraclass correlation coefficients (ICCs) (one-way random effects model) (40). The assessment of the level of reliability was based on Shrout's recommendations: (41) an ICC of 0.21 to 0.4 indicate “slight,” an ICC of 0.41 to 0.60 indicate “fair,” an ICC of 0.61 to 0.80 indicate “moderate,” and an ICC of 0.81 to 1.00 indicate “substantial.”

Kappa values and 95% confidence intervals (CI) were calculated to determine the degree of agreement between the data from VSA-3000 and tuning fork, VSA-3000 and SSEP, tuning fork and SSEP, respectively. Kappa values were used to test agreement between sets of results, which vary between 0 and 1 (0–0.50: slight to moderate agreement; 0.51–0.60: acceptable agreement; 0.61–0.80: substantial agreement; 0.81–1.00: almost perfect agreement) (42).

The sensitivity (ability of the test to correctly identify proprioception impairment), specificity (ability of the test to correctly identify proprioception spared), positive predictive value (proportion of positive test results that were from proprioception impaired patients), and negative predictive value (proportion of negative test results that were from proprioception spared patients) of VSA-3000 and tuning fork tests were calculated, using the results of SSEP tests as the criteria, and presented with 95% CI.

To compare the diagnostic accuracy of two types of tests against the reference standard of SSEP measurement, receiver operating characteristic (ROC) curve were constructed for each test (43), using the full range of possible thresholds per test. Areas under the receiver operating characteristic curve (AUC) are a measure of the performance of a test in predicting the outcome of interest. Generally, AUC values of 0.5 indicate that a test performs no better than chance, values between 0.70 and 0.79 indicate fair performance, values between 0.80 and 0.89 indicate good performance, and values ≥0.9 indicate excellent test performance (10). Statistical significance of the difference between the AUCs were tested with the method of DeLong et al. (44).

Stepwise multiple linear regression analysis (45) was used to examine the relationship between VPTs and age, height, gender, groups (stoke or SCI), types, and locations of injury of the patients. Types of injury were assessed by replacing types with dummy variables (cerebral hemorrhage, cerebral infarction, complete SCI, or incomplete SCI). Likewise, locations of injury were assessed by replacing locations with dummy variables (basal ganglia, SCI of thoracic levels, or SCI of lumbar levels and cauda equina injury and conus and cauda equina injury).

All analyses were performed using the version of SPSS 17.0. The significance level was set at *P* < 0.05.

## Results

### Data of VPTs and Tuning Fork

We described the VPTs measured with VSA-3000 (according to the age groups) and the tuning fork scores in Table 3. Four sites per one patient for 30 stroke patients and two sites per one patient for 30 SCI patients, therefore, in total 60 participants with 180 sites of data.

**Table 3:** VPTs measured with VSA-3000 and tuning fork scores.

	**Age (yrs)**	***n***	**Middle finger**		**Great toe**	
			**Left**	**Right**	**Left**	**Right**
VPTs	20–29	12	8.33 ± 8.02	3.2 ± 2.97	70 ± 62.87	69.36 ± 63.59
(μm)	30–39	12	102.73 ± 23.69	1.3 ± 0.46	85.83 ± 56.56	68.53 ± 64.23
	40–49	14	26.81 ± 43.94	24.1 ± 46.06	57.91 ± 56.75	52.67 ± 60.08
	50–59	17	37.27 ± 47.93	24.75 ± 47.22	66.26 ± 54.52	47.97 ± 48.68
	60–69	3	24.8 ± 31.68	65.9 ± 90.65	93.77 ± 62.76	88.5 ± 71.88
	71	2	5.8	130	15.2 ± 12.45	67.85 ± 87.89
Tuning fork	20–71	60	6.47 ± 3.85	10 (10, 10)	4 (0, 10)	9 (0, 10)

*Values are mean ± standard deviation or median (P25, P75)*.

### Test-Retest Reliability

Participants in the reliability of the study completed two identical VSA-3000 test sessions with ~1 to 4 weeks between each session (mean interval = 15.7 days) (Table 4). The VPTs showed substantial reliability as the ICC is 0.91 (95% CI, 0.88~0.93).

**Table 4:** VPT results of test–retest.

**Test**	**Patients**	**Middle finger**		**Great toe**	
		**Left**	**Right**	**Left**	**Right**
First	Stroke (*n* = 30)	12.35 (2.30, 64.33)	2.55 (1.38, 19.05)	23.70 (15.25, 130.00)	11.90 (6.20, 64.55)
	SCI (*n* = 30)	NT	NT	130.00 (12.78, 130.00)	130.00 (10.58, 130.00)
Second	Stroke (*n* = 30)	32.02 ± 39.20	3.55 (1.80, 10.70)	52.63 ± 44.49	14.00 (6.43, 60.48)
	SCI (*n* = 30)	NT	NT	105.10 (12.20, 130.00)	125.35 (8.23, 130.00)

*NT, not tested. Values are mean ± standard deviation or median (P25, P75)*.

### Consistency

With regard to the test results of tuning fork and VSA-3000, the kappa value was 0.731 (95% CI: 0.647 to 0.815, *P* < 0.001) (Table 5), indicating that the consistency of the two test results was not good enough.

**Table 5:** Consistency for tuning fork and VSA-3000.

**Tuning fork**	**VSA-3000**			**Total**
	**Normal**	**Decreased**	**Undetected**	
10	76	6	2	84
1–9	7	28	11	46
0	0	5	45	50
Total	83	39	58	180

When the consistency between the test results of VSA-3000 and SSEP was examined, the kappa value was 0.877 (95% CI: 0.806 to 0.948, *P* < 0.001) (Table 6), indicating that there was nearly perfect agreement between the two test results.

**Table 6:** Results for 180 sites that were tested with VSA-3000 to distinguish between proprioception impaired and spared.

Results of VSA-3000^*^	**SSEP results**		**Total**
	**Proprioception impaired**	**Proprioception spared**	
Positive	91	6	97
Negative	5	78	83
Total	96	84	180

* *A positive result indicates a VPT of VSA-3000 test is decreased or undetected, and a negative result indicates a VPT is normal*.

Level of consistency between the test results of tuning fork and SSEP (kappa value, 0.732; 95% CI, 0.632 to 0.832; *P* < 0.001) (Table 7) was much lower than that between VSA-3000 and SSEP, which suggested that the test results of VSA-3000 were much closer to the SSEP test results than that of tuning fork.

**Table 7:** Results for 180 sites that were tested with tuning fork to distinguish between proprioception impaired and spared.

Results of tuning fork^*^	**SSEP results**		**Total**
	**Proprioception impaired**	**Proprioception spared**	
Positive	84	12	96
Negative	12	72	84
Total	96	84	180

* *A positive result indicates a score of tuning fork <10, and a negative result indicates a score of tuning fork = 10*.

### Validity

The VSA-3000 test had a sensitivity (i.e., its ability to correctly detect proprioception impaired patients) of 94.8% (95% CI, 87.7 to 98.1%; Table 6) and a specificity (i.e., ability to correctly detect proprioception spared patients) of 92.9% (95% CI, 84.5 to 97.1%). The positive-predictive value of VSA-3000 (i.e., correctly identifying a proprioception impaired patient) was 93.8% (95% CI, 86.5 to 97.5%) and the negative-predictive value (i.e., correctly identifying a proprioception spared patient) was 94.0% (95% CI, 85.9 to 97.8%).

The tuning fork test had a sensitivity of 87.5% (95% CI, 78.8 to 93.1%) and a specificity of 85.7% (95% CI, 76.0 to 92.1%; Table 7), which were both lower than that of VSA-3000. The positive-predictive value was 87.5% (95% CI, 78.8 to 93.1%) and the negative-predictive value was 85.7% (95% CI, 76.0 to 92.1%), which were also lower than VSA-3000.

**Figure 5:**
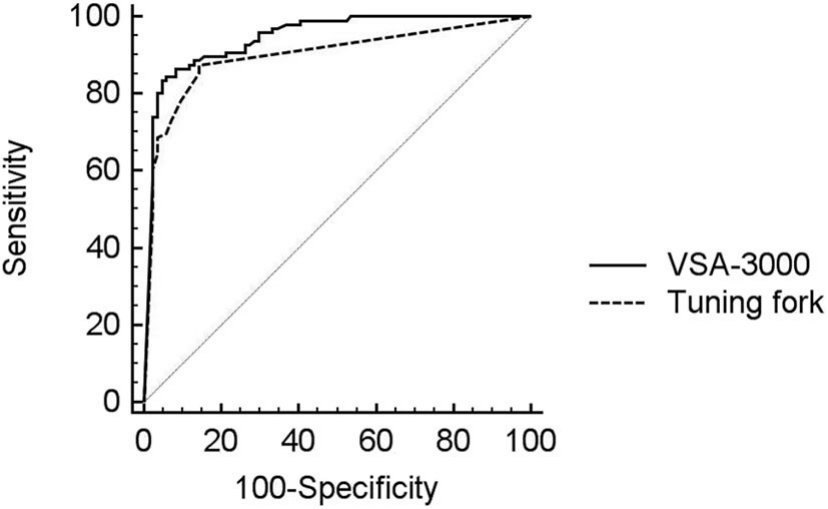
ROC curve analysis was used to evaluate the diagnostic performance of VSA-3000 (black line) and tuning fork (dashed line) in detecting proprioception perception impairment against SSEP results using the evoked potential instrument. The gray line is the null value of the ROC curve.

The diagnostic performance of the VSA-3000 and tuning fork (using continuous VPT outputs and tuning fork scores) in detecting proprioception perception impairment against the SSEP test results is given in the AUC using ROC curve analysis (Figure 5). The AUC for VSA-3000 is 0.95 (SE: 0.017, 95% CI: 0.91 to 0.98, *P* < 0.001) and the AUC for tuning fork is 0.89 (SE: 0.025, 95% CI: 0.85 to 0.95, *P* < 0.001). The diagnostic accuracy of VSA-3000 was significantly better than that of tuning fork (*P* = 0.0216 < 0.05).

### Relationship Between VPTs and Demographic Characteristics

To investigate the relationship between VPTs and demographic characteristics of subjects, we performed a stepwise multiple regression analysis (n = 60) with VPTs as the dependent variables and age, height, gender, groups (stoke or SCI), types and locations of injury of the patients as independent variables. The types of injury (complete SCI vs. other types) and age were significantly related to VPTs (*R*^2^ = 0.389, *P* < 0.001). None of the other factors significantly added to the model. Regression results are shown in Table 8.

**Table 8:** Multiple regression analyses predicting VPTs for participants with stroke and SCI.

**Multiple regression analysis**	**β**	***t*-value**	***p*-value**
**Variables in model**			
Types of injury (Complete SCI vs. Other types)	0.652	10.545	<0.001
Age	0.130	2.094	0.038
**Variables not in model**			
(Constant)	__	0.768	0.444
Height	0.049	0.766	0.444
Gender	−0.023	−0.392	0.695
Groups	0.004	0.048	0.962
Types of injury (Cerebral hemorrhage vs. Other types)	0.048	0.659	0.511
Types of injury (Cerebral infarction vs. Other types)	−0.072	−1.203	0.230
Locations of injury (SCI of thoracic levels vs. Other locations)	−0.049	−0.556	0.579
Locations of injury (SCI of lumbar levels and cauda equina injury and conus and cauda equina injury vs. Other locations)	0.029	0.483	0.630

*Types of injury: cerebral hemorrhage, cerebral infarction, complete SCI, or incomplete SCI; Groups: stroke or SCI; Locations of injury: basal ganglia, SCI of thoracic levels, or SCI of lumbar levels and cauda equina injury and conus and cauda equina injury*.

## Discussion

The present study determined the use of VSA-3000 as potential diagnostic testing instrument for patients with CNS injury. Specifically, our primary aims were to determine the diagnostic accuracy of VSA-3000 against the reference standard of SSEP and to evaluate the superior accuracy compared with tuning fork in patients with CNS injury. Although the sample size was relatively small, this study provides preliminary support for the reliability and validity and the superior of this methodology in persons with stroke and SCI.

### Reliability

In our sample of individuals with stroke and SCI, the test-retest reliability of threshold measures for vibratory detection showed substantial reliability (0.91). This result is consistent with studies in healthy, non-disabled subjects and in other patient populations (31, 46). A study by Felix and Widerström-Noga examined vibration thresholds across two test sessions in a sample of SCI patients with neuropathic pain and a sample of non-disabled control subjects, and the results showed that the ICCs were in the substantial range (0.86–0.90) (25). Two other studies have remarked on the stability of VPTs obtained in persons with SCI (24, 47). Krassioukov et al. found that the ICC in incomplete SCI patients for VPT was in the range 0.76–0.90 (24). A recent article aimed to determine the psychometric properties of the Graph-DCK Scale in people with SCI and neuropathic pain, involving detection of VPT in the test procedures, noted that ICCs for VPT were 0.83 and 0.85 for at-level assessment and below-level assessment, respectively (47). The previous studies results agree with our results, suggesting reasonable reliability of VPTs between sessions in patients with stroke and SCI.

### Consistency

The high kappa value between VSA-3000 and SSEP reported in this study (0.877) indicated that there was excellent consistency between the two test results, which was higher than that between tuning fork and SSEP (0.732). Therefore, compared with tuning fork, the test results of VSA-3000 showed a higher degree of similarity to SSEP test results.

Some previous studies have focused on the relationships between VTPs and other measurements. Hayes et al.'s study found significant kappa values (denoted by κ) obtained from incomplete SCI patients for the association between VPT and light touch values for the right L4 (κ = 0.25) and left L4 (κ = 0.29) dermatomes and also a significant correlation between VPT and pinprick for the right L4 dermatome (κ = 0.33) (6). In addition, Santos et al. investigated the relationship between VPT and neuropathic signs of patients with type two diabetes, and found a clear trend toward progressively greater VPT in patients with mild and moderate/severe signs in contrast to patients with absent neuropathic signs (12).

### Validity

In addition to the examination of reliability and consistency of VPTs in persons with stroke and SCI, a preliminary analysis of the validity of VSA-3000 test as diagnostic and outcome measures was also examined.

The present study used the sensitivity, specificity, positive- and negative-predictive values and ROC curves, as used by Martin et al. (10), to evaluate the utility of VPT to predict proprioception impairment.

The sensitivity and negative-predictive value of VPT obtained in our study compared favorably to Martin et al.'s study (sensitivities between 72 and 93% and negative-predictive values between 58 and 91%), however, the specificities (47–63%) and positive-predictive values (37–80%) of Martin et al.'s were lower than our study (10). The ROC curves demonstrate the clear tradeoff between sensitivity and specificity when VPT is used as a predictor of proprioception impairment. The areas under the ROC curve (AUC) suggest that VPT performance is excellent (0.95), which is higher than Martin et al.'s study of using VPT as a measure of distal symmetrical peripheral neuropathy in type 1 diabetes (0.71–0.83) (10). Two other studies have showed fairly good predictive performance of VPT. Santos et al. found the AUC of VPT for detection of diabetic peripheral neuropathy (DPN) in patients with type two diabetes was 0.71 (12), and Pritchard et al.'s study showed the AUC for diagnosis of 4-year incident DPN in type 1 diabetes was 0.74 (14).

The discrepancies between previous studies and our study may be attributed to applying in different type of diseases. The previous studies investigated VPT as a measure of peripheral neuropathy, and found VPT might provide important, clinically meaningful information about large nerve fiber dysfunction in diabetes (10). The present study used the VPT as a measure of proprioception impairment in central nervous system injury, in relation to electrophysiological testing (SSEP) as reference standard, as both measures are believed to reflect integrity of the dorsal columns (6).

In addition, the sensitivity, specificity, positive- and negative-predictive values and ROC curve of tuning fork were also evaluated, which were all lower than VPT. These results suggest that the degree of validity of tuning fork in persons with CNS diseases is similar to that seen in other patient populations. We noticed that Arshad and Alvi's study (48) showed that the tuning fork test, in patients with type 2 diabetes, had high specificity (93.70%), but low sensitivity (55.88%), the positive- and negative-predictive value were 70.37 and 88.81%, respectively, and the AUC for tuning fork is 0.75.

### Relationship Between VPTs and Demographic Characteristics

Results from the multiple linear regression analysis in the present study suggest that the types of injury (complete SCI vs. other types) and age may significantly influence the VPTs, regardless of the height, gender, groups and locations of injury.

We show that the types of injury (complete SCI vs. other types) were the factor highly correlated with the VPTs. The possible reason for this result may be that most of the complete SCI patients had no sensation and therefore would artificially increase the correlations as the tests showed absent responses. Although Felix and Widerström-Noga's study showed the severity of injury (complete vs. incomplete) was not significantly related to Neuropathic Pain Symptom Inventory total intensity score (25), the participants of their study were SCI-related neuropathic pain and the relationship they investigated was between somatosensory thresholds and severity of neuropathic pain symptoms.

The current report indicates that vibratory thresholds changed linearly with age, which is not unexpected. Association between age and VPT has been previously shown in general populations (31, 49), and in diabetic patients (10, 12, 50). Many factors may contribute to decline of vibration sensitivity, such as age-related reduction in the receptor density, morphological modifications of the remaining receptors, and possible degeneration of corresponding peripheral nerves fibers (12).

Since height is highly correlated with latencies of cortical SSEPs (51–53), we include height in our methodology. Previous studies had reported that VPT, especially measured at the lower extremity, positively correlated with height (31, 54–60). However, we found no significant correlation between height and VPT in our patients with CNS injury. A possible cause for such interesting issue is that subject heights in our group were normally distributed with a standard deviation of only 6.49 cm; therefore, very few participants lay far from the mean to give strength to an analysis of height in this context (61). Although the lack of correlation should not be over interpreted in this relatively small sample, our findings are consistent with some studies in healthy subjects and in other patient populations that showed similar results (61–65).

All the results from this analysis should be viewed with a modest degree of caution, as the data available for this analysis were relatively small (*n* = 60). Although most other variables included in the regression analysis (height, gender, groups, and locations of injury) displayed non-significant relationships with the dependent variable, the lack of a mediating effect of these variables is inconclusive as a result of the low power.

### Limitations and Future Research

The present study must be interpreted in the context of its potential limitations. We use latencies of SSEP, rather than amplitudes, as the reference criteria based on a consideration that “latencies seem to be more reliable in reflecting real damage, whereas amplitudes vary inter-individually and depend more on the quality of the peripheral nerve stimulation” (37). However, some studies have been made to analyze both latencies and amplitudes for different research purposes (66, 67). The multiple linear regression analysis in the present study showed that the types of injury (complete SCI vs. other types) and age were the factor highly correlated with the VPTs, especially complete vs. incomplete spinal lesions. Studies in the past addressed this situation by either excluding complete SCI patients from the study or at least stratifying the population, with and without complete SCI correlations (6, 68). Therefore, the findings in this study need to be replicated in a larger study to further detail the reliability and validity of VSA-3000 test in people with stroke and SCI, and the amplitudes of SSEP will be incorporated in the evaluations and the SCI population will be stratified, with and without complete SCI correlations, in the future works.

## Conclusion

Based on the results of the present study, VSA-3000 appears to provide a reliable and accurate assessment of impaired vibration sensation caused by central nerve injury. Use of VSA-3000 as a diagnostic and/or outcome measurement strategy may provide new motivations for its applications in the clinic and large-scale clinical trial researches.

## Clinical Messages

1. VSA-3000 has good diagnostic accuracy for assessing impaired vibration sensation caused by central nerve injury.
2. VSA-3000 is a non-invasive and convenient QST instrument that may provide a new method to quantitatively test vibration sense in clinic.

## Data Availability Statement

All datasets generated for this study are included in the article/Supplementary Material.

## Ethics Statement

The studies involving human participants were reviewed and approved by Ethical Committee of the China Rehabilitation Research Center. The patients/participants provided their written informed consent to participate in this study.

## Author Contributions

All authors listed have made a substantial, direct and intellectual contribution to the work, and approved it for publication.

## Conflict of Interest

The authors declare that the research was conducted in the absence of any commercial or financial relationships that could be construed as a potential conflict of interest.
