# Supplementary material for: VSA-3000: A Quantitative Vibration Sensation Testing Device for Patients With Central Nervous System Injury
Source: Front Neurol. 2020 Sep 8;11:936. doi: 10.3389/fneur.2020.00936 (PMC7505990; doi:10.3389/fneur.2020.00936)
Supplement: Supplementary file 1 [file Table_1.pdf]

VSA-3000: A quantitative vibration sensation testing device for patients with central nervous system injury.

Appendix

Table A: The VPT results of stroke patients

Patient No.	Sex	Age(yrs)	Height(cm)	Diagnosis	Course of disease(d)	Left middle finger	Left middle finger-retest	Right middle finger	Right middle finger-retest	Left great toe	Left great toe-retest	Right great toe	Right great toe-retest
1	Female	48	160	CH，L	28	43.2	60.2	3.5	4.3	41.1	33.6	16.2	21.8
2	Female	53	158	CH，L	30	84.2	56.7	0.9	2.1	54.3	72.4	12.2	10.7
3	Male	35	172	CH，L	97	87.2	46.9	1.4	1.6	59	48.5	6.4	7
4	Male	44	172	CH，R	47	1.3	1.5	2.9	4.6	4.7	3.9	6.6	10
5	Male	57	163	CI，L	34	10.8	15.4	3	3.7	15.8	7.6	22.3	18.7
6	Male	41	167	CH，R	22	1.4	1.6	50.7	62.3	6.6	7.3	130	89
7	Male	41	170	CH，L	151	9.5	7.3	1.3	1.6	30	15.3	5.2	3
8	Female	33	168	CH，L	230	130	130	0.8	1.1	130	96.8	5.9	5.2
9	Male	59	170	CH，R	95	1.9	1.5	23.4	17.8	11.1	8.9	45.9	69.5
10	Male	57	170	CH，R	176	3.1	3.5	130	89	14.2	15	130	67
11	Male	36	180	CH，L	29	91	67	1.7	1.8	130	102.6	5.6	6.2
12	Female	59	160	CH，L	107	57.7	38.9	1.8	2.3	130	121.5	15.4	20.8
13	Male	52	170	CH，R	78	3.9	4.2	17.6	9.4	17.7	29.4	37	58.3
14	Male	51	180	CH，L	98	130	76.6	1	2.3	130	118.7	15.2	10.4
15	Male	49	176	CH，L	119	9.8	2.6	1.1	1.6	130	116.8	6.9	17.1
16	Male	53	170	CH，R	34	2	1.6	130	14.6	21.2	11.3	130	68.2
17	Male	49	170	CH，R	35	1.7	1.8	130	130	1.4	1.5	130	99.5
18	Male	29	168	CH，R	58	1.6	1.9	6.6	8.7	5.1	4.6	11.2	48.7
19	Male	48	170	CH，L	81	130	120.4	1.5	2.3	130	73.8	5.5	5
20	Male	29	177	CH，L	44	17.2	36.1	1.9	1.8	21.4	41.2	5.2	5.8
21	Female	71	152	CH，R	38	5.8	6.3	130	86.7	24	32.5	130	107.4
22	Female	44	167	CH，L	15	17.6	2.5	1.8	1.8	19.4	99.8	6.3	8.7
23	Male	52	175	CH，L	58	130	130	2.2	3.8	130	98.7	11.6	10.9
24	Male	56	165	CI，L	144	13.9	20.7	4.5	3.9	15.6	38.3	120.5	34.2
25	Male	60	178	CH，R	38	2.4	2.1	130	130	21.3	17.8	130	126
26	Male	51	176	CH，L	32	26.2	46.7	3.3	3.5	45.3	56.2	22.4	31.1
27	Male	29	180	CH，L	70	6.2	6.6	1.1	1.5	11.3	17.2	5.4	3
28	Male	62	163	CH，L	108	47.2	19.7	1.8	2.4	130	130	5.5	3.2
29	Male	52	170	CH，L	35	1.6	17.6	1.1	0.9	23.4	27.8	8	7.9
30	Male	52	170	CH，L	82	19.2	32.7	3	3.6	116.4	130	7.9	6.5

Note: all patients with lesions in basal ganglia, CH=cerebral hemorrhage, CI=cerebral infarction, L(R): left(right) hemiplegia

Table B: The VPT results of spinal cord injury (SCI) patients

Patient No.	Sex	Age(yrs)	Height(cm)	Diagnosis	Course of disease(d)	etiologies	Left great toe	Left great toe-retest	Right great toe	Right great toe-retest
1	Male	38	170	cauda equina injury	113	fall	40.9	66.8	10.8	16.8
2	Male	38	177	T5 incomplete SCI	269	vertebral hemangiomatosis	5.1	12.4	4.5	5.7
3	Female	35	163	T7 incomplete SCI	67	vehicle crash	6.1	5.3	130	130
4	Male	22	178	cauda equina injury	299	fall	1.8	2.3	1.4	1.8
5	Male	27	170	L2 complete SCI	121	fall	130	130	130	130
6	Male	55	168	T4 complete SCI	24	fall	130	118.9	130	130
7	Male	21	173	L2 incomplete SCI	56	fall	15.4	22.5	24.6	46.7
8	Male	27	173	T12 complete SCI	20	violence	130	130	130	130
9	Male	21	175	T12 complete SCI	23	vehicle crash	130	130	130	130
10	Female	22	163	T10 incomplete SCI	38	vehicle crash	5	5.9	4.5	6.2
11	Male	35	162	T10 incomplete SCI	43	fall	8.9	4.5	9.1	7.8
12	Female	39	155	T6 complete SCI	64	vehicle crash	130	130	130	130
13	Female	62	160	T8 complete SCI	66	fall	130	130	130	130
14	Male	31	175	T8 complete SCI	33	fall	130	107.8	130	122.4
15	Male	33	165	conus and cauda equina injury	74	fall	130	130	130	130
16	Male	37	173	T8 complete SCI	20	violence	130	102.4	130	128.3
17	Male	49	168	T11 complete SCI	32	violence	130	130	130	130
18	Male	46	170	T10 complete SCI	30	violence	130	130	130	130
19	Male	47	175	cauda equina injury	20	violence	29.9	62.4	26.4	38.9
20	Male	51	168	conus and cauda equina injury	275	vehicle crash	11.5	15.2	15.3	6.8
21	Male	20	178	T3 complete SCI	25	fall	130	130	130	130
22	Male	49	173	T9 complete SCI	69	violence	130	130	130	130
23	Male	35	173	T3 complete SCI	83	fall	130	98.3	130	67.4
24	Male	21	174	T12 complete SCI	35	fall	130	130	130	130
25	Male	70	160	T12 incomplete SCI	127	no obvious cause	6.4	5.8	5.7	8.3
26	Male	27	172	T10 complete SCI	41	fall	130	130	130	130
27	Male	51	182	T9 incomplete SCI	46	spinal cord compression from vertebral hemangioma	130	47.8	77.6	8
28	Male	44	178	T7 incomplete SCI	72	viral encephalomyelitis	14.4	11.6	4.4	4.9
29	Male	40	170	cauda equina injury	80	violence	13.2	10.6	9.9	10
30	Male	58	170	L2 incomplete SCI	85	fall	130	130	14.2	13.7

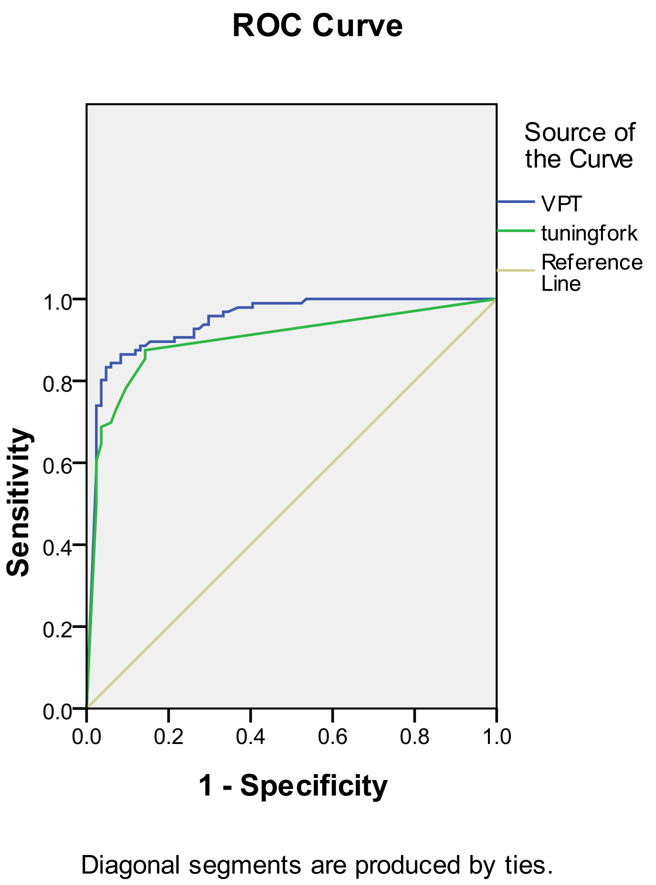

Fig A. ROC curve generated by SPSS
